# Resistance Mechanisms of Anti-PD1/PDL1 Therapy in Solid Tumors

**DOI:** 10.3389/fcell.2020.00672

**Published:** 2020-07-21

**Authors:** Qingyang Lei, Dan Wang, Kai Sun, Liping Wang, Yi Zhang

**Affiliations:** ^1^Biotherapy Center, The First Affiliated Hospital of Zhengzhou University, Zhengzhou, China; ^2^Cancer Center, The First Affiliated Hospital of Zhengzhou University, Zhengzhou, China; ^3^Henan Key Laboratory for Tumor Immunology and Biotherapy, Zhengzhou, China; ^4^College of Medicine, Zhengzhou University, Zhengzhou, China; ^5^School of Life Sciences, Zhengzhou University, Zhengzhou, China

**Keywords:** cancer, immunotherapy, PD1, PDL1, resistance, mechanism

## Abstract

In cancer-immunity cycle, the immune checkpoint PD1 and its ligand PDL1 act as accomplices to help tumors resist to immunity-induced apoptosis and promote tumor progression. Immunotherapy targeting PD1/PDL1 axis can effectively block its pro-tumor activity. Anti-PD1/PDL1 therapy has achieved great success in the past decade. However, only a subset of patients showed clinical responses. Most of the patients can not benefit from anti-PD1/PDL1 therapy. Furthermore, a large group of responders would develop acquired resistance after initial responses. Therefore, understanding the mechanisms of resistance is necessary for improving anti-PD1/PDL1 efficacy. Currently, researchers have identified primary resistance mechanisms which include insufficient tumor immunogenicity, disfunction of MHCs, irreversible T cell exhaustion, primary resistance to IFN-γ signaling, and immunosuppressive microenvironment. Some oncogenic signaling pathways also contribute to the primary resistance. Under the pressure applied by anti-PD1/PDL1 therapy, tumors experience immunoediting and preserve beneficial mutations, upregulate the compensatory inhibitory signaling and induce re-exhaustion of T cells, all of which may attenuate the durability of the therapy. Here we explore the underlying mechanisms in detail, review biomarkers that help identifying responders among patients and discuss the strategies that may relieve the anti-PD1/PDL1 resistance.

## Introduction

Cancer immunotherapy has achieved great clinical advances over the past few years, especially in the fields of adoptive cell transfer therapy and the application of immune checkpoint blockade (ICB). Among those developing and inspiring immunotherapies, ICB targeting programmed cell death 1/programmed cell death ligand 1 (PD1/PDL1) axis is famous for the Nobel Prize in 2018 and has been approved for use in different solid tumors.

Antigen-specific T cells play pivotal roles in the process of eliminating tumor cells. Tumor cells trigger the cancer-immunity cycle once they release tumor antigens (Chen and Mellman, 2013). Antigen-specific T cells initially undergo recognitions of tumor antigens presented by major histocompatibility complexes (MHCs) on antigen presenting cells (APCs), and subsequently experience priming and activation. After activation and proliferation, T cells travel to the particular sites following chemokine concentration gradient. For an optimal activation, except that costimulatory signal transductions are required between T cells and APCs, immune checkpoints like PD1 and its ligand PDL1 are also needed to avert the excessive activation of T cells (Chen and Mellman, 2013; Maimela et al., 2018). However, researchers have found that tumors accommodate themselves to resisting the apoptosis induced by immune system in multiple ways. One tactic tumors use to escape immune rejection is to take advantage of immune checkpoints (Pardoll, 2015).

**FIGURE 1:**
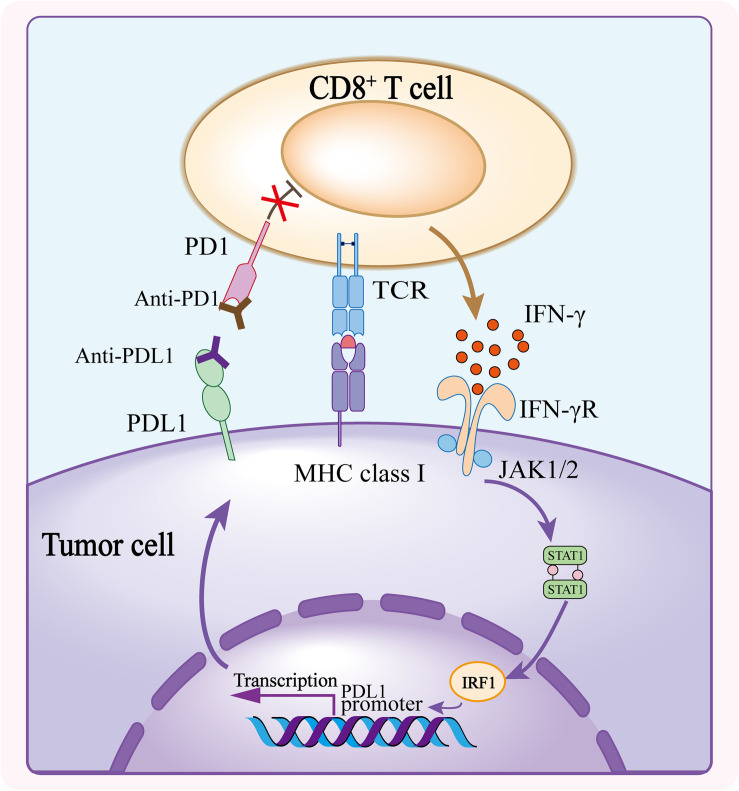
Mechanism of PD1/PDL1 blockade. The CD8^+^ T cell activates upon recognizing the tumor antigen presented on MHC class I and releases IFN-γ to bind to IFN-γ receptor, and consequently induces the expression of PDL1 on tumor cells. PDL1 conjugates the elevated PD1 on T cell surface, triggering inhibitory effect of PD1/PDL1 axis. Anti-PD1 or anti-PDL1 antibody blocks the interaction of PD1 and PDL1, and abolishes the inhibition of CD8^+^ T cell thus enhancing the antitumor activity.

Upon confronting the same antigen on MHCs, T cells release IFN-γ to enhance efficiency of tumor killing. The release of IFN-γ from CD8^+^ T cells upregulate the expression of PDL1 on tumor cells and stromal cells (Ribas, 2015; Garcia-Diaz et al., 2017). Meanwhile, TCR signaling upregulates the expression of PD1 on T cell surface which binds to PDL1 to exert negative regulatory effects and blunt the antitumor function of T cells (Agata et al., 1996; Ribas and Wolchok, 2018; Akinleye and Rasool, 2019). Immunotherapy against the interaction between PD1 and PDL1 reinvigorates T cells that were inactive because of the PD1/PDL1 signaling inhibition. It has been proved that PD1/PDL1 blockade significantly enhances antitumor effects in different solid tumors including melanoma, non–small cell lung cancer (NSCLC), renal cell carcinoma, head and neck squamous cell carcinoma, urothelial carcinoma, microsatellite instability–high colorectal cancer etc., and the list is still growing (Ansell et al., 2015; Motzer et al., 2015; Robert et al., 2015; Chow et al., 2016; Kaufman et al., 2016; Bellmunt et al., 2017; El-Khoueiry et al., 2017; Overman et al., 2017; Powles et al., 2017; Sharma et al., 2017b; Fuchs et al., 2018; Akinleye and Rasool, 2019; Chung et al., 2019; Figure 1).

However, clinical data of anti-PD1/PDL1 therapy showed limited response rates. Previous investigations revealed that a large group of patients suffered primary resistance and did not respond to PD1/PDL1 blockade, and some of the responders developed acquired resistance after initial responses as well (Sharma et al., 2017a). More disturbingly, the underlying mechanisms largely remain unknown.

In this review, we will focus on documented mechanisms of primary and acquired resistance of anti-PD1/PDL1 therapy and summarize the biomarkers that can potentially discriminate responders from non-responders. Then we will discuss the development of strategies for enhancing the antitumor efficacy and overcoming the resistance of anti-PD1/PDL1 therapy.

## Primary Resistance

**FIGURE 2:**
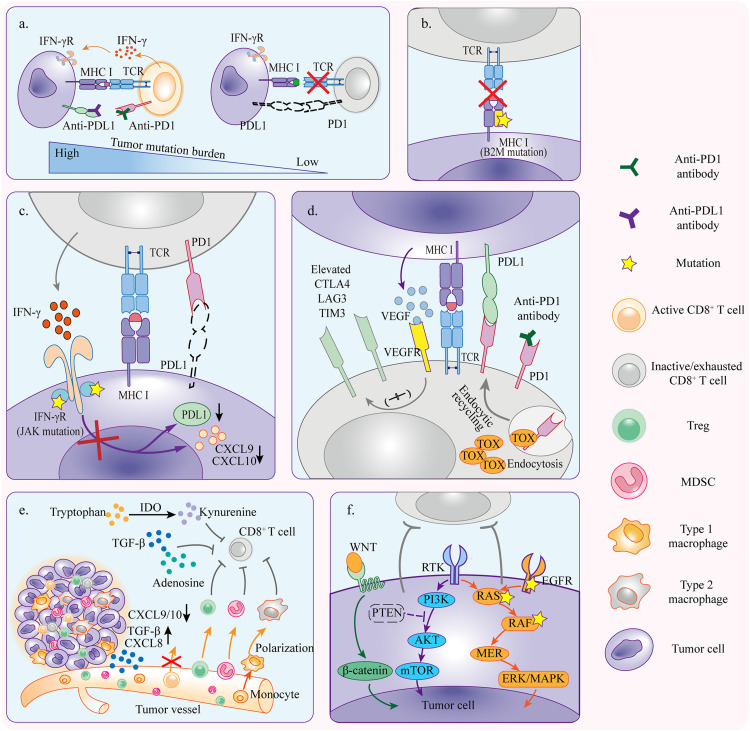
Mechanisms of primary resistance. **(a)** Tumors with high mutation burden are more likely to respond to anti-PD1/PDL1 therapy because there is a greater possibility of generating immunogenic neoantigens which activate CD8^+^ T cells and induce tumor rejection reactivity. **(b)** Tumor cells with primary B2M mutation fail to present tumor antigens and elicit antitumor immunity which is required for tumor cell killing. **(c)** Tumor cells resistant to IFN-γ signaling because of primary JAK1/2 mutation can not induce PDL1 upregulation but inhibit T cell reactivity in PD1/PDL1 independent pathway. The inactivation of IFN-γ signaling also downregulates CXCL9 and CXCL10 expressions which are necessary for T cell trafficking. **(d)** Alternative immune checkpoints are upregulated in tumor infiltrating T cells, and thus only blocking PD1/PDL1 axis is not enough to rescue the severely exhausted T cells. Upregulation of VEGFR signaling and TOX expression aggravates the activation of inhibitory signaling. **(e)** TME contains diverse immunosuppressive cells that influence anti-PD1/PDL1 efficacy through inhibiting T cell reactivity. Cytokines derived from tumors recruit more immunosuppressive cells to TME and promote polarization toward pro-tumor phenotype. **(f)** Oncogene mutation and abnormal activation contribute to inhibition of antitumor immunity, resulting in primary resistance to anti-PD1/PDL1 therapy.

Despite the fact that the immunotherapy against cancer PD1/PDL1 axis is becoming more feasible, the limited efficacy of anti-PD1/PDL1 therapy was observed in patients with different solid tumors. Anti-PD1/PDL1 therapy works through strengthening the functions of preexisting CD8^+^ T cells (Tumeh et al., 2014; Tang et al., 2016). Nonetheless, in anti-PD1/PDL1 therapy, tumors can escape tumor rejection by shaping a hostile tumor microenvironment (TME) to impede the antitumor efficacy of T cells. This may occur due to insufficient antigen immunogenicity, disfunction of antigen presentation, irreversible T cell exhaustion, resistance of IFN-γ signaling, and immunosuppressive TME (Figure 2).

### Insufficient Tumor Immunogenicity

The antitumor efficacy of PD1/PDL1 blockade depends on the existence of antigen-specific T cell reactivity in TME, which requires potential tumor rejection antigens presented by dendritic cells (DCs) to cross-priming CD8^+^ T cells and triggering subsequent antitumor activity (Mempel et al., 2004). Theoretically, there are two classes of antigens capable of inducing antitumor immunity; one is formed by non-mutated proteins with incomplete T cell tolerance and another one is formed by mutated proteins which are known as neoantigens (Heemskerk et al., 2013). Deep sequencing technology has helped to identify non-synonymous mutations which are able to generate neoantigens in tumors (Schumacher and Schreiber, 2015). Exome sequencing of two independent cohorts of NSCLC patients who have received anti-PD1 therapy showed that the non-synonymous mutational burden was higher in patients with durable clinical benefit (Rizvi et al., 2015). Further analysis of neoantigen landscape demonstrated that it was the candidate neoantigen burden which reflected the quantity of putative HLA class epitopes, rather than frequency of non-synonymous mutation that related to anti-PD1 response (Rizvi et al., 2015). Coincidentally, tissues derived from melanoma patients who were sensitive to PD1 blockade harbored more non-synonymous single nucleotide variants and were detected with higher putative HLA class I and class II neoepitope loads compared with those from non-responders’ (Hugo et al., 2016). Extrapolating from those evidences, we can speculate that tumors with high mutational burden have higher probability to generate more neoantigens with adequate immunogenicity capable of inducing antigen-specific T cell reactivity. Based on the evidence that the infiltration of CD8^+^ T cells is higher in tumors with higher mutational burden, neoantigens are thought particularly relevant to antitumor immunity (Brown et al., 2014). Moreover, cancers deficient in mismatch repair genes harbored high numbers of mutations that would generate neoantigens, making them sensitive to anti-PD1 therapy despite of different tissue origins (Le et al., 2017). On the contrary, tumors with low mutational burdens, such as prostate and pancreatic cancer, are more likely not to respond to therapy, due to the lack of immunogenic neoantigens (Schumacher and Schreiber, 2015).

### Disfunction of MHCs

Antigen presentation mainly occurs through MHC class I pathway in TME, and thus tumors can escape T cell killing by inactivating MHC class I complex. B2M is essential for assembly and stabilization of HLA class I complex (Hulpke and Tampé, 2013). Hypothetically, disfunction of HLA class I due to B2M mutation causes antigen-presenting malfunction, resulting in the attenuation of T cell cytotoxicity. Indeed, an investigation of tumor tissues illustrated that B2M mutations were frequently present in CRC with microsatellite instability, indicating the existence of B2M mutation in pre-therapy MSI-H CRC (Yeon Yeon et al., 2019). Further, a more direct evidence revealed that loss of heterozygosity (LOH) at B2M locus happened three folds higher in non-responders compared with responders from a cohort of melanoma patients treated with PD1 blockade (Sade-Feldman et al., 2017). Similar phenomenon was also observed in an anti-CTLA4 treated cohort, proposing B2M mutation as a common mechanism of primary ICB resistance. Collectively, tumors may resist to anti-PD1/PDL1 therapy by disturbing MHC class I function with B2M mutation.

### Irreversible T Cell Exhaustion

T cell exhaustion has been viewed as a dysfunctional state upon long-lasting existence of tumor antigens and immunosuppressive TME (Wherry and Kurachi, 2015). Considering that PD1/PDL1 blockade works by reinvigorating impaired cytotoxic T cells, mechanisms that engender irreversible T cell exhaustion probably contribute to anti-PD1/PDL1 resistance (McLane et al., 2019; Zhang Z. et al., 2020). Exhausted CD8^+^ T cells express multiple inhibitory receptors, encompassing PD1, T-cell immunoglobulin and mucin-domain containing 3 (TIM3), lymphocyte-activation gene 3 (LAG3) and cytotoxic T lymphocyte-associated antigen 4 (CTLA4). It is supposed that anti-PD1/PDL1 antibodies can only surmount part of the suppressive signaling in TME, but there are more inhibitory axes that preclude the function of T cells in the TME. Exactly, analysis of tissues from NSCLC patients elucidated that TIM3 was upregulated on T cell surface, resulting in anti-PD1 resistance (Limagne et al., 2019). In addition, high amount of tumor-derived VEGF induced high level of PD1, CTLA4, TIM3, and LAG3 expression which were makers of exhaustion on the surface of CD8^+^ T cells, contributing to resistance in anti-PD1 treatment (Voron et al., 2015). Importantly, the amount of PD1 expression on T cell surface also interferes the efficacy of anti-PD1 therapy. T cells that express high level of PD1 along with multiple inhibitory receptors from NSCLC patients had poor functional recovery after anti-PD1 therapy (Thommen et al., 2015). Severely exhausted CD8^+^ T cells that express high level of PD1 do not respond to PD1 blockade and, instead, T cells with intermediate expression level of PD1 is responsive to anti-PD1 therapy (Blackburn et al., 2008; Wherry, 2011). Thymocyte selection-associated high mobility group box gene (TOX) was reported as a positive regulator of PD1, TIM3, TIGIT, and CTLA4 expression in the tumor infiltrating CD8^+^ T cells, regulating CD8^+^ T cell exhaustion. The expression level of TOX on tumor infiltrating CD8^+^ T cells was negatively correlated with anti-PD1 response rates in melanoma and NSCLC patients (Kim et al., 2020). TOX was reported maintaining the PD1 expression on T cell surface by mediating endocytic recycling of PD1, and overexpression of TOX exacerbated exhaustion of CD8^+^ T cells, which could hinder the CD8^+^ T cells from responding to anti-PD1 therapy (Khan et al., 2019; Wang et al., 2019).

### Resistance of IFN-γ Signaling

IFN-γ produced by T cells upon tumor antigen recognition signals through IFN-γ receptor and consequently results in expression of IFN-γ stimulated genes by inducing activation of Janus kinase JAK1/2, and phosphorylation of the signal transducers and activators of transcription (STAT) (Bach et al., 1997; Platanias, 2005). Most of the stimulated genes benefit to antitumor immunity, while others like *CD274* (encoding PDL1) lead to inactivation of tumor-specific T cells (Ribas, 2015). Mutations of JAK1/2 disrupt the IFN-γ signaling transduction and lead to paucity of PDL1 expression. Despite high tumor mutational burden (TMB) being often considered as a marker of responsive anti-PD1/PDL1 therapy, studies revealed that the resistance of PD1/PDL1 blockade in some high-mutated tumors was probably attributed to the JAK1/2 mutations. Researchers analyzed samples from melanoma and colon cancer patients who were tested having a high TMB, yet did not respond to PD1 blockade therapy (Shin et al., 2017). They found that those patients had homozygous loss-of-function mutations in JAK1/2, which led to deficiency of PDL1 expression even in the presence of IFN-γ, making it fruitless to block PD1 and PDL1 interaction. Moreover, the JAK1/2 controls expression of chemokines (e.g., CXCL9, CXCL10, and CXCL11) which are potent to attract T cells. Therefore, it was rational that tumors with loss-of-function mutations of JAK1 were indeed short of T-cell infiltration (Shin et al., 2017).

### Immunosuppressive Microenvironment

Tumor cells educate surrounding environment to suppress antitumor immunity and support their proliferation, differentiation, expansion, and invasion. Immunosuppressive cells, cytokines and tumor metabolites in the microenvironment restrain antitumor efficacy (Gajewski et al., 2013; Li X. et al., 2019). Regulatory T cells (Tregs) act as negative mediators of antigen-specific T cell function, which gives the privilege to tumors for escaping the antitumor immunity (Tanaka and Sakaguchi, 2017). Tregs suppress activation, proliferation and functions of CD8^+^ T cells through generating immunosuppressive substances such as IL-10, TGF-β and extracellular adenosine, depriving IL-2 in TME, and constitutively expressing CTLA4 (Tanaka and Sakaguchi, 2017). Increased infiltration of Tregs in tumors is correlated with poor prognosis (Sasada et al., 2003; Curiel et al., 2004; Bates et al., 2006). *In vivo* studies showed that Tregs which induced high level of PD1 expression in CD8^+^ T cells were responsible for the primary anti-PD1 resistance (Ngiow et al., 2015).

Myeloid-derived suppressive cells (MDSCs) are a group of immature myeloid cells with suppressive competence in tumor microenvironment. MDSCs consist of two large groups of cells: granulocytic or polymorphonuclear MDSCs (PMN-MDSCs) and monocytic MDSCs (M-MDSCs). MDSCs produce immunosuppressive factors including but not limited to ROS, NO, and IL-10, through which can suppress both antigen-specific and non-specific T cell response, and instigate tumor invasion and angiogenesis (Marvel and Gabrilovich, 2015; Veglia et al., 2018). Besides, it is reported that the increased galectin-9^+^ M-MDSC in peripheral blood of NSCLC patients is involved in resistance of anti-PD1 therapy (Limagne et al., 2019). Thereby, the presence of MDSCs in TME is detrimental for anti-PD1/PDL1 response. As expected, several studies revealed the relationship between MDSCs infiltration and PD1 blockade resistance, and selective depletion of MDSCs could restore the anti-PD1 efficacy (Highfill et al., 2014; De Henau et al., 2016).

Tumor associated macrophages (TAMs) are theoretically divided into two phenotypes: M1 macrophages and M2 macrophages. TAMs, especially those belonging to M2 phenotype, are considered to suppress functions of CTL, recruit immunosuppressive cells and promote tumor progression through secreting inhibitory cytokines and generating other suppressive factors (Yang and Zhang, 2017). Clinical studies identified a correlation between TAMs accumulation and poor clinical outcomes. Therefore, targeting TAMs is expected to induce tumor regression (Yang and Zhang, 2017; Zhou et al., 2020). Presence of TAMs in pancreatic cancer exaggerates immunosuppression within microenvironment and leads to the PD1/PDL1 blockade resistance. Inhibition of colony-stimulating factor 1 receptor (CSF1R) on TAMs can upregulate the expression of PDL1 and increase CD8^+^ T cell infiltration, which ablates anti-PD1/PDL1 resistance (Zhu et al., 2014).

Cytokines are key modulators in TME mediating recruitment and polarization of immune cells. For example, transforming growth factor beta (TGF-β) plays a multifaceted role in TME. TGF-β promotes tumor progression by inducing epithelial-mesenchymal transition of tumor cells, recruiting immunosuppressive cells like Tregs and MDSCs as well as inhibiting functions of CD8^+^ T cells (Batlle and Massagué, 2019). Studies found that TGF-β was associated with poor clinical outcomes and limited the response of anti-PDL1 therapy which was attributed to T cell exclusion in urothelial and colorectal cancer (Mariathasan et al., 2018; Tauriello et al., 2018). TGF-β1, the universal isoform of TGF-β, presents in many human cancers and contributes to anti-PD1 resistance (Martin et al., 2020). Tumor-derived chemokines recruit immunosuppressive cells including Tregs, macrophages and MDSCs to the TME to create an immunosuppressive but tumor-supportive environment, interfering the immunotherapy targeting adaptive immune resistance (Roussos et al., 2011). Elevated CXCL8 expression in tumors recruited MDSCs or M2-like TAMs that disrupted the efficacy of anti-PD1 (Highfill et al., 2014; Zhang M. et al., 2020). Furthermore, tumor-derived chemokines not only regulate suppressive cell recruitment, but also prevent T cell infiltration. Epigenetic modulation involves in silencing Th1 type chemokines such as CXCL9 and CXCL10, and thereby impeding anti-PD1/PDL1 efficacy by mediating T cell trafficking to the tumor site (Peng et al., 2015).

Indoleamine 2,3-dioxygenase (IDO) catalyzes tryptophan (TRP) catabolism to generate kynurenine (KYN). In TME, IDO creates an immunosuppressive climate through suppressing the functions of T cells while promoting generation and activation of Tregs and MDSCs (Prendergast et al., 2014). In soft tissue sarcoma treatment, the upregulation of IDO may cause anti-PD1 resistance. After the combination therapy of pembrolizumab and metronomic cyclophosphamide, the increased infiltration of IDO^+^ M2 failed the therapy because tumor shrinkage was observed in just 3 of the patients with only one experiencing partial response when the total number was 57 and over 40% patients expressed PDL1 in the TME (Toulmonde et al., 2018).

As a consequence of oxygen deprivation, adenosinergic pathway activates in tumors and induces the extracellular adenosine accumulation. CD39 and CD73 are two important enzymes for converting ATP into adenosine (Vijayan et al., 2017). Redundant extracellular adenosine has been identified as an inhibitor of the antitumor activity, which directly binds to A2A receptor on T cell surface or favors polarization of immune cells toward immunosuppressive phenotypes (Cekic et al., 2014; Vijayan et al., 2017). Consistently, preclinical studies demonstrated an enhancement of anti-PD1 efficacy when combining anti-PD1/PDL1 therapy with treatment of A2A receptor antagonist (Beavis et al., 2015; Willingham et al., 2018).

### Classic Oncogene Mutation

Studies have demonstrated that oncogenic signaling plays a pivotal role in the formation of immunosuppressive TME and promoting tumor immune escape (Spranger and Gajewski, 2016; Yang et al., 2019). The contribution of tumor-intrinsic oncogenic pathways for resistance of PD1/PDL1 blockade should not be overlooked.

EGFR is an important molecular phenotype of NSCLC. Study found that EGFR mutation rates were negatively correlated with PDL1 expression which is necessary for PD1/PDL1 blockade (Ji et al., 2016). A retrospective analysis elucidated that patients with EGFR and ALK mutation had poor response rates when treated with anti-PD1/PDL1 therapy. Intriguingly, patients with EGFR mutation had lower PDL1 expression level than others with ALK rearrangement. The concurrent existence of PDL1 expression and high levels of CD8^+^ tumor infiltrating lymphocyte (TILs) were only present in a small group of EGFR mutant tumors, suggesting an underlying mechanism of anti-PD1/PDL1 resistance in patients with EGFR or ALK mutation (Gainor et al., 2016).

NSCLC patients with KRAS mutation were reported more responsive to anti-PD1/PDL1 therapy. The mechanism is that KRAS mutation promotes infiltration of CD8^+^ T cells and increases TMB and tumor immunogenicity (Liu et al., 2020). However, another study revealed that KRAS mutation in tumors can cause anti-PD1 resistance by repressing IRF2 expression which correspondingly promoted the recruitment of MDSC through increasing CXCL3 expression (Liao et al., 2019). Combining PD1 blockade with IRF2 overexpression or CXCR2 (CXCL3 receptor) blockade relieved anti-PD1 resistance (Liao et al., 2019). Moreover, co-mutation in KRAS and STK11/LKB1 contributed to a lower objective response rate in lung adenocarcinoma treated with nivolumab (Skoulidis et al., 2018).

Mitogen-activated protein kinase (MAPK) pathway mediates multiple cellular processes, such as proliferation, apoptosis, and migration. The aberration of MAPK pathway may give rise to carcinogenesis (Dhillon et al., 2007). Alteration of the Ras-MAPK pathway inhibits the recruitment and infiltration of T cells in triple negative breast cancer (Loi et al., 2016). Expectedly, preclinical studies demonstrated intensified efficacy of PD1/PDL1 blockade when combined with MAPK-targeted therapy (Hu-Lieskovan et al., 2015; Hugo et al., 2016; Loi et al., 2016).

Phosphatase and tensin homolog (PTEN) loss results in activation of the phosphatidylinositol-3-kinase (PI3K) pathway and consequently causes tumorigenesis (Keniry and Parsons, 2008). Evidence showed that patients with metastatic uterine leiomyosarcoma which uniquely harbored biallelic PTEN loss were resistant to anti-PD1 therapy (George et al., 2017). PTEN loss in tumor cells leads to inhibition of T cell function by secreting VEGF as well as recruiting immunosuppressive cells. Additionally, elevated expression of immunosuppressive cytokines reduced the infiltration of T cells in tumor sites (Peng et al., 2016).

Wnt/β-catenin signaling is essential for embryonic development and tissue homeostasis (MacDonald et al., 2009). Dysregulation of Wnt/β-catenin signaling pathway was observed promoting multiple carcinogenesis (Zhan et al., 2017). The abnormal activation of WNT/β-catenin signaling pathway was responsible for the impaired naïve T cell priming in melanoma (Spranger et al., 2015). *In vivo* study revealed that the β-catenin signaling downregulated CCL4 expression, which led to the defective recruitment of DCs to the tumor (Spranger et al., 2015).

## Acquired Resistance

**FIGURE 3:**
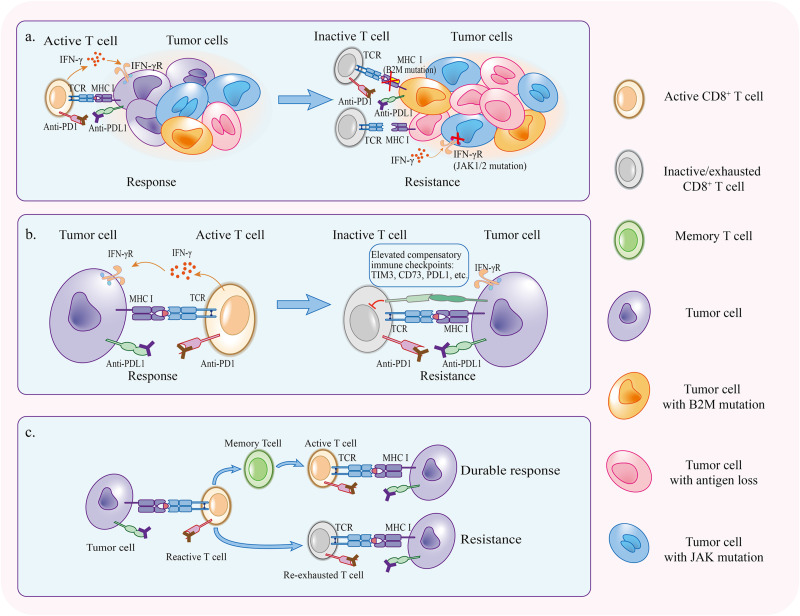
Mechanisms of acquired resistance. **(a)** Immunoediting under the pressure applied by PD1/PDL1 blockade selectively preserves tumor cells that have superiority to escape antitumor immunity. **(b)** Compensatory inhibitory signaling is upregulated in the course of therapy, making it difficult for PD1/PDL1 blockade to reinvigorate CD8^+^ T cells. **(c)** If tumor specific T cells fail to become memory T cells, therapy response will not persist and T cells will be re-exhausted.

Some patients will develop resistance or relapse eventually after the initial response in the treatment with PD1/PDL1 blockade. The host immune system sometimes is an accomplice of therapy resistance. During the course of anti-PD1/PDL1 therapy, tumor cells which enable to escape from antitumor immunity gradually occupied a predominant proportion via cancer immunoediting (O’Donnell et al., 2019). In addition, the activation of PD1/PDL1 independent inhibitory pathways and re-exhaustion of reinvigorated T cells can retard the function of T cells again in the presence of PD1/PDL1 blockade (Figure 3).

### Immunoediting of Tumor Subclones

The concept of immunoediting suggests that the immunity boosted by anti-PD1/PDL1 therapy not only protects the host against tumor progression but also results in selection of tumor subclones that can escape antitumor immunity, which ends up leading to the acquired resistance of anti-PD1/PDL1 therapy (Schreiber et al., 2011; Matsushita et al., 2012; O’Donnell et al., 2019). Antigen loss due to DNA copy-number alterations in tumor cells was observed in the presence of antigen-specific CTLs producing IFN-γ, indicating genetic changes of neoantigens under the pressure of tumor-specific immunity (Takeda K. et al., 2017). Neoantigens generated from non-synonymous mutation play an important role in inducing intratumoral T cell response. Immunoediting that gives rise to neoantigen loss may result in acquired anti-PD1/PDL1 resistance. An analysis of matched pretreatment and posttreatment tumor tissues derived from patient who had developed acquired resistance confirmed that the loss of 7 to 18 putative mutation-associated neoantigens, a consequence of genomic alteration, was detected in tumors with acquired resistance (Anagnostou et al., 2017). Further study found that the eliminated neoantigens were capable of eliciting clonal T cell expansion in autologous T cell culture, suggesting the T-cell-dependent immunoselection occurred under the pressure of PD1/PDL1 blockade (Anagnostou et al., 2017).

Although the B2M mutation has been observed in pre-immunotherapy samples and was partly responsible for primary resistance mechanisms of anti-PD1/PDL1 therapy, more proofs exemplified that tumors including melanoma, CRC and lung cancer acquired B2M mutations in the course of the treatment and were resistant to PD1/PDL1 blockade consequently (acquired resistance) (Zaretsky et al., 2016; Gettinger et al., 2017; Sade-Feldman et al., 2017; Yeon Yeon et al., 2019). Researchers investigated a series of specimen from biopsy of baseline, regressive disease and progressive disease of one melanoma patient who has received sequential treatment of anti-CTLA4 and anti-PD1 antibodies, and samples from anti-PD1 responders and non-responders as well (Sade-Feldman et al., 2017). They found that the LOH and upstream frameshift mutations in B2M were only extensively present during disease progression (Sade-Feldman et al., 2017), suggesting immunoediting of B2M-mutated tumor subclones has already existed earlier in tumor development, or became dominant under selective pressure applied by ICBs. Similar results have also been verified in other studies (Zaretsky et al., 2016; Gettinger et al., 2017; Le et al., 2017; Pereira et al., 2017).

In the setting of patients who developed acquired resistance after continuous anti-PD1/PDL1 therapy, tumors that were no longer sensitive to IFN-γ may survive and grow. Homozygous mutation of JAK1/2 was detected in post-treatment samples collected from melanoma patients who had tumor progression after initial response, suggesting clonal selection and proliferation of JAK-inactive tumor cells after applying the PD1 blockade (Zaretsky et al., 2016).

### Compensatory Inhibitory Signaling

As was reviewed before, immunotherapy targeting PD1 or PDL1 only blocks one of many immune checkpoints, but there are still chances that tumors would escape immune elimination by activating additional inhibitory signaling or elevating PDL1 expression. A subset of melanoma expressed MHC II and was reported sensitive to anti-PD1 therapy (Johnson et al., 2016). However, another immune checkpoint LAG3 was upregulated on TILs in MHC II^+^ tumors in the failure of the anti-PD1 treatment after initial response (Johnson et al., 2016, 2018). In preclinical and clinical studies, the upregulation of TIM3 was detected on TILs after treatment, subsequently leading to acquired resistance in PD1/PDL1 blockade (Koyama et al., 2016; Oweida et al., 2018; Limagne et al., 2019). Furthermore, tumors that developed resistance to anti-PD1/PDL1 therapy were found accompanied with upregulation of CD73 expression or higher serum kynurenine/tryptophan ratio, indicating the presence of metabolic alteration in the mechanism of acquired resistance that limits the persistence of anti-PD1/PDL1 efficacy (Reinhardt et al., 2017; Li H. et al., 2019). Additionally, inhibitory signaling derived from suppressive TME may abolish the efficacy of PD1/PDL1 blockade. The reactivation of CD8^+^ T cells consequently induced the PDL1-NLRP3 inflammasome signaling cascade and eventually led to acquired resistance due to the accumulation of MDSCs (Theivanthiran et al., 2020).

### Re-exhaustion of T Cells

Following effectivity exertion and antigen clearance, a small group of effector T cells transform into memory cells that will be reactivated and expand when reencountering tumor antigens (Harty and Badovinac, 2008). Effector memory T cells were observed predominantly increasing in responders during anti-PD1 therapy (Ribas et al., 2016). If the high level of antigen expression keeps maintained and the exhausted T cells reinvigorated by PD1/PDL1 blockade fail to become memory T cells, the durability of reinvigoration will be limited after the therapy. A study of mouse model of chronic lymphocytic choriomeningitis virus (LCMV) infection found that the exhausted T cells acquired an epigenetic profile which was distinct from memory T cells and became re-exhausted after persistent anti-PDL1 treatment, resulting in relapse which demonstrated the similar viral load compared with control (Pauken et al., 2016).

## Predictive Biomarkers

Despite the impressive success anti-PD1/PDL1 treatment achieved, the primary and acquired resistance still occurs in most of solid tumor patients. Predictive biomarkers are urgently needed to guide the treatment selection, sequentially reducing the economic burden and improving the survival of patients. According to understanding of the underlying mechanisms of PD1/PDL1 blockade, potential biomarkers basically include those which demonstrate immunogenic antigen landscape and preexisting immune context (Ren et al., 2020).

In consideration of the matter that adaptive immunity activation is the basics for PD1/PDL1 blockade, a favorable antigen recognition is required (Tumeh et al., 2014). The antigen recognition may be influenced by the immunogenicity of neoantigens that are closely relevant to TMB and MSI. High TMB and MSI involve in increased total number of neoantigens that may generate more immunogenic neoantigens which induce strong immune response (Schumacher and Schreiber, 2015). Concomitantly, TMB reflecting tumor mutational frequency has been used as an indicator in clinical settings; unresectable solid tumors with MSI-H or dMMR has been approved by FDA as one of the indications of pembrolizumab treatment (Goodman et al., 2017; Cristescu et al., 2018; Marcus et al., 2019). However, biomarkers associated with mutational load are not perfect because the existence of tumor-specific antigen is just one of many necessary conditions for driving the cancer-immunity cycle, and only a tiny part of neoantigens can be presented by MHC and recognized by TCR (Schumacher and Schreiber, 2015; Yi et al., 2018).

The presence of CD8^+^ T cells in TME is a prerequisite for anti-PD1/PDL1 therapy. Accordingly, the density of TILs is positively correlated with PD1/PDL1 blockade efficacy (Fridman et al., 2012). Measuring TILs by immunohistochemistry (IHC) might be promising but still needs to be standardized. PDL1 expression within tumors has been identified as “canary in a coalmine,” because it proves the existence of T cells (Ribas and Wolchok, 2018). PDL1 expression detected by IHC has been widely used as predicator in multiple cancers (Topalian et al., 2012; Garon et al., 2015; Carbone et al., 2017). However, the reliability of using IHC to detect PDL1 expression within tumor is imperfect (Patel and Kurzrock, 2015). The systemic PDL1 expression in exosome has been explored recently as a potential predictive biomarker for identifying responders (Daassi et al., 2020). Additionally, patients bearing PDL1-positive tumors are not always responsive to anti-PD1/PDL1 therapy. In contrast, PDL1-negative tumors are not meant for anti-PD1/PDL1 resistance (Gibney et al., 2016). Thus, it is not appropriate to regard PDL1 expression as a “yes or no” biomarker. In a pilot study, researchers found that majority of NSCLC patients who were not responsive to anti-PD1 therapy despite with high PDL1 expression were detected having high galectin-3 expression within tumors, suggesting a synergistic use of certain biomarkers may be superior (Capalbo et al., 2019).

Emerging evidence showed that the diversity and composition of the gut microbiota can influence anti-PD1/PDL1 efficiency (Inamura, 2020). Ayelet and colleagues first noticed that the differences among tumor bearing mice rely on distinction of commensal microbiota (Sivan et al., 2015). Oral administration of *Bifidobacterium* can promote the efficacy of anti-PDL1 antibody in mice (Sivan et al., 2015). In patients, commensal microbial composition was found associated with clinical anti-PD1/PDL1 response rate, and fecal microbiota transplantation from responders into germ-free or antibiotic-treated mice leveraged the restraint of PD1 blockade (Gopalakrishnan et al., 2018b; Matson et al., 2018).

## Strategies for Overcoming Resistance

Following development of research, strategies targeting different steps of tumor immune escape have been brought up. Analogous to the “whack a mole” game, unless anti-PD1/PDL1 therapy is combined with other strategies, tumors may pop up at any defenseless step of cancer-immunity cycle. In this case the combination therapies are needed for achieving better clinical outcomes (Song et al., 2018). Currently, strategies mainly focus on enhancing T cell priming, reversing T cell exhaustion, increasing T cell infiltration and improving the immunosuppressive microenvironment (Table 1).

**TABLE 1:** Strategies for overcoming anti-PD1/PDL1 resistance.

Mechanism	Type of strategy	Example	Phase	Tumor type	Effect	Trial number	References
Enhancing T cell priming	Chemotherapy	Pembrolizumab plus carboplatin and paclitaxel	III	Metastatic squamous NSCLC	Improving overall survival and progression-free survival	NCT02775435	Paz-Ares et al., 2018
		Pemetrexed and a platinum-based drug plus pembrolizumab	III	Metastatic non-squamous NSCLC	Improving overall survival and progression-free survival	NCT02578680	Gandhi et al., 2018
	Oncolytic virus	T-VEC plus pembrolizumab	III	Stage IIIB-IV melanoma	Ongoing	NCT02263508	Long et al., 2016
	vaccine	Multi-peptide vaccine plus nivolumab	I	HLA-A*0201 positive, HMB-45, NY-ESO-1, and/or MART-1 positive resected tumors	Tolerated	NA	Gibney et al., 2015
		Neoantigen vaccine plus pembrolizumab	I	Stage IIIB/C and IVM1a/b melanoma	Tumor regression	NCT01970358	Ott et al., 2017
	Radiotherapy	Previous radiotherapy plus pembrolizumab	I	NSCLC	Longer progression free and overall survival	NCT01295827	Shaverdian et al., 2017
	TLRs agonist	TLR3-specific RNA agonist (ARNAX) plus anti-PDL1 antibody	*In vivo* experiments	EG7 cell line	Tumor regression	NA	Takeda Y. et al., 2017
		TLR9 agonist lefitolimod plus anti-PD1 or anti-PDL1 antibody	*In vivo* experiments	A20 and CT26 cell lines	Tumor regression	NA	Kapp et al., 2019
	IFN-α	IFN-α-anti-PDL1 fusion protein	*In vivo* experiments	A20, MC38, B16F10, and L929 cell lines	Tumor regression	NA	Liang et al., 2018
Reversing T cell exhaustion	Other ICBs	Anti-TIM3 blocking antibody plus Nivolumab	*In vitro* experiments	NSCLC	Reversing resistance to anti-PD-1 in PBMC from lung cancer patients	NCT02281214	Limagne et al., 2019
		Anti-TIGIT plus anti-PD1 antibody	*In vivo* experiments	GBM	Improving overall survival	NA	Hung et al., 2018
	Costimulatory agonist	Agonistic anti-CD40 antibody plus anti-PD1 antibody	*In vivo* experiments	BALB/c Renca renal carcinoma	Downregulating PD-1 expression Tumor regression	NA	Ngiow et al., 2016
Increasing T cell infiltration	Costimulatory agonist	Antibody-guided LIGHT fusion protein plus anti-PDL1 antibody	*In vivo* experiments	C57BL/6 AT3 mammary adenocarcinoma	Increasing T cell infiltration Tumor regression	NA	Tang et al., 2016
Improving immunosuppressive microenvironment	TGF-β blockade	TGF-β blockade plus anti-PDL1 antibody	*In vivo* experiments	EMT6 and MC38 cell lines	Facilitating T-cell penetration Tumor regression	NA	Mariathasan et al., 2018
Improving the immunosuppressive microenvironment	TGF-β blockade	TGF-β1 blockade SRK-181-mIgG1 plus anti-PD1 antibody	*In vivo* experiments	EMT6, Cloudman S91 and MBT2 cell lines	Facilitating T-cell penetration Tumor regression	NA	Martin et al., 2020
	Chemokine/cytokine receptor blockade	CSF1R blockade plus anti-PD-1 antibody	*In vivo* experiments	Pancreatic ductal adenocarcinoma	Tumor regression	NA	Zhu et al., 2014
		Anti-CCR4 mAb	*In vitro* experiments	Melanoma	Depletion of effector Tregs	NA	Sugiyama et al., 2013
		Anti-CXCR2 mAb plus anti-PD1 antibody	*In vivo* experiments	Mouse rhabdomyosarcoma	Tumor regression	NA	Highfill et al., 2014
	PI3K inhibitor	Selective PI3K inhibitor plus anti-PD1 antibody	*In vivo* experiments	B16 cell line	Tumor regression	NA	De Henau et al., 2016
	Epigenetic modulators	DZNep and 5-AZA-dC plus PD1 blockade	*In vivo* experiments	Moue ovarian cancer	Slowing down tumor progression	NA	Peng et al., 2015
	IDO inhibitor	IDO inhibitor INCB23843 plus anti-PDL1 and anti-CTLA4 antibody	*In vivo* experiments	B16 cell line	Tumor regression	NA	Spranger et al., 2014
	Adenosinergic pathway inhibitor	A2AR antagonist ciforadenant plus anti-PDL1 antibody	*In vivo* and *in vitro* experiments	MC38 cell line	Tumor regression Improving survival	NA	Willingham et al., 2018
		A2AR antagonist ciforadenant plus atezolizumab	I	Renal cell carcinoma	Tumor regression	NCT02655822	Fong et al., 2020
		CD73 inhibitor MEDI9447 plus anti-PD1 antibody	*In vitro* and vivo experiments/I	Mouse 4T1 cell line and human MDA-MB-231 cell line	Tumor regression in preclinical study	NCT02503774 (Ongoing)	Hay et al., 2016
Combination with other therapies	Oncogenic pathway inhibitor	BRAF inhibitor vemurafenib plus anti-PD1 or anti-PDL1 antibody	*In vivo* experiments	Mouse BRAF mutated tumor	Tumor regression Prolonged survival	NA	Cooper et al., 2014
		Dabrafenib and trametinib plus pembrolizumab	I	BRAF ^*V600*^-mutated metastatic melanoma	Durable response	NCT02130466	Ribas et al., 2019
	Commensal microbiota	Oral administration of *Bifidobacterium* plus anti-PDL1 antibody	*In vivo* experiments	Melanoma	Tumor regression	NA	Sivan et al., 2015
		Fecal transplant plus anti-PDL1 antibody	*In vivo* experiments	Melanoma	Tumor regression	NA	Matson et al., 2018

### Enhancing T Cell Priming

Chemotherapy can promote tumor antigen release and provide damage-associated signals upon inducing tumor apoptosis, converting DCs to immunostimulatory APCs for CD8^+^ T cell priming (Grimaldi et al., 2020). For example, decitabine can enhance subsequent antigen recognition through upregulating MAGE-A3 expression in esophageal carcinoma (Shi et al., 2019). Some chemotherapeutic drugs, like gemcitabine and cyclophosphamide, simultaneously deplete MDSC and Tregs while killing tumor cells (Suzuki et al., 2005; van der Most et al., 2009). As expected, studies demonstrated synergistic effects in patients receiving combination of chemotherapy and PD1/PDL1 blockade (Zitvogel et al., 2011; Gandhi et al., 2018; Paz-Ares et al., 2018; Grimaldi et al., 2020).

Clinical studies in melanoma patients displayed improved overall survival when combining anti-PD1 therapy with multi-peptide vaccine which improved T cell priming by increasing antigen presentation (Gibney et al., 2015). Intriguingly, some patients showed complete response in synergistic treatment of personalized tumor neoantigen vaccination and anti-PD1 therapy (Ott et al., 2017). Similar to self vaccination, oncolytic virus therapy also induces tumor antigen release and provides danger signals, which consequently enhances T cell priming and ameliorates anti-PD1/PDL1 resistance (Guo et al., 2014; Long et al., 2016). Radiation therapy upregulates type I interferon production by inducing tumor cell death that activates STING pathway and promotes T cell priming (Twyman-Saint Victor et al., 2015; Darragh et al., 2018). NSCLC patients who receive previous radiotherapy showed a prolonged survival after pembrolizumab treatment (Shaverdian et al., 2017).

Additionally, the regimen of targeting TLRs, such as TLR3, TLR9, promotes DCs maturation and rationally alleviates the anti-PD1/PDL1 resistance (Takeda Y. et al., 2017; Kapp et al., 2019). Since the upregulation of CTLA4 expression has been considered as a negative regulator in mediating T cell priming, the combination therapy of anti-PD1 and anti-CTLA4 antibodies significantly extended the patients’ overall survival (Wolchok et al., 2017). Furthermore, type I IFNs are critical for T cell priming (Deng et al., 2014). In a preclinical study, researchers designed an anti-PDL1-IFN-α fusion protein to specifically deliver IFN-α into tumors. Systemic administration of the fusion protein achieved complete regression in most of the tumor bearing mice (Liang et al., 2018). Collectively, strategies enhancing T cell priming can improve the sensitivity of anti-PD1/PDL1 therapy.

### Reversing T Cell Exhaustion

The expressions of coinhibitory immune checkpoint receptors upregulate following T cell activation. Strategies targeting alternative immune checkpoints or amplifying costimulatory signals may contribute to the relief of anti-PD1/PDL1 resistance (Marin-Acevedo et al., 2018). Synergistic therapy of PD1/PDL1 blockade and other ICBs including TIM3 and TIGIT blockade have been proved for harboring superior survival outcomes in several studies (Hung et al., 2018; Limagne et al., 2019). In addition, agonistic antibody targeting CD40 has been identified significantly reversing T cell exhaustion, which was illuminated by reactivated T cells with augmented production of cytokines and capacities of cytotoxicity and proliferation (Ngiow et al., 2016).

### Increasing T Cell Infiltration

Sufficient T cell infiltration is a prerequisite for reinvigorating antitumor immunity through PD1/PDL1 blockade. In a preclinical study, taking tumor bearing mice treated with anti-EGFR-guided LIGHT fusion protein into account, the study illustrated that the activation of lymphotoxin β-receptor induced production of chemokines which led to extensive T cell recruitment. The combination therapy with PDL1 blockade significantly results in tumor regression (Tang et al., 2016). Furthermore, combination therapy of PD1/PDL1 blockade with adoptive cell transfer increases the infiltration and cytotoxicity of T cells, which breaks the restriction of MHC disfunction (Blake et al., 2015).

### Improving the Immunosuppressive Microenvironment

Immunosuppressive microenvironment inhibits the function of the main force of antitumor immunity i.e., CD8^+^ T cells, resulting in anti-PD1/PDL1 resistance. Targeting TAMs accumulated in TME can effectively improve response to the therapy (Cassetta and Kitamura, 2018). Combination treatment of CSF1R blockade with anti-PD1 antibody reprogrammed M2 into M1 and promoted tumor regression in tumor bearing mice (Zhu et al., 2014). Immunosuppressive cytokines derived from tumors not only attract immune cell infiltration and drive their polarization into pro-tumor phenotype, but also directly inhibit CD8^+^ T cell cytotoxicity (Lippitz, 2013). Based on this mechanism, targeting cytokines especially TGF-β promotes T cell penetrating and augments antitumor immunity (Mariathasan et al., 2018), and selective blockade of the most prevalent isoform TGF-β1 can significantly reduce the dose-dependent side effect while enhancing anti-PD1 efficacy (Martin et al., 2020). Moreover, desensitizing chemokine receptors by inhibitors prevents the infiltration of immunosuppressive cells including MDSCs and Tregs, which ultimately increases sensitivity of tumors to PD1/PDL1 blockade (Sugiyama et al., 2013; Highfill et al., 2014). PI3K-γ was reported highly expressed in myeloid cells. Selective inhibition of PI3K-γ shifted myeloid cells to a less suppressive phenotype and increased CD8^+^ T cell infiltration, hence spurred the efficacy of anti-PD1 therapy (De Henau et al., 2016). Epigenetic silencing of Th1 type chemokines such as CXCL9 and CXCL10, involves in immunosuppression. Epigenetic modulation through DNA methyltransferase and histone deacetylase inhibitors rationally sensitizes tumors to anti-PD1/PDL1 therapy (Peng et al., 2015).

Metabolites play pivotal roles in modulating tumor immunosuppressive context. IDO has been shown to influence the TME by converting tryptophan to kynurenine. Evidence has documented that IDO inhibitors combined with anti-PDL1 and anti-CTLA4 antibodies enhanced proliferation and infiltration of CTLs and IL2 production in preclinical models (Spranger et al., 2014). However, precise biomarker may be needed for selecting responders because there is no significant benefit but side effects that lead to discontinuation of several clinical trials (Günther et al., 2019).

Adenosine is a product of adenosinergic pathway. Accumulation of adenosine in TME abrogates antitumor immunity. Complete tumor elimination has been observed in up to 90% mice treated with antagonist of A2A receptor accompanied by anti-PDL1 or anti-CTLA4 antibody (Willingham et al., 2018). Targeting adenosine production by inhibiting the enzyme CD73 efficaciously facilitated anti-PD1/PDL1 efficiency (Hay et al., 2016; Chen et al., 2019). Recently, the first-in-human study of A2AR antagonist treatment has been conducted in renal cell carcinoma patients with anti-PD1/PDL1 resistance (Fong et al., 2020). Clinical response was observed when treated anti-PD1/PDL1 resistant patients with either A2AR antagonist alone or combining with anti-PDL1 antibody (Fong et al., 2020).

### Combination With Other Therapies

Combination of PD1/PDL1 blockade with other therapies has been widely investigated. Therapies targeting the oncogenic signaling that can influence the TME by mediating cytokine production, bringing benefits to anti-PD1/PDL1 therapy. Take the inhibitors targeting MAPK pathway as an example. In preclinical study, targeting BRAF mutation resulted in an increase in CD8^+^ T cell infiltration and IFN-γ production (Cooper et al., 2014). Concurrently, triple combination therapy inhibiting BRAF, ERK and PD1 in patients with BRAF V600-mutated metastatic melanoma displayed persistent antitumor response (Ribas et al., 2019). In addition, the influence of composition of gut microbiota in host immunity makes it not only a predicator, but also a target for combination therapy. Strategies including dietary modification, probiotics administration and fecal microbiota transplantation have made progress in preclinical research and still need further investigation in clinical applications (Gopalakrishnan et al., 2018a).

## Conclusion

The application of PD1/PDL1 blockade has become a milestone of immunotherapy. Although the anti-PD1/PDL1 therapy has shown impressive efficacy in the treatment of solid tumors, the durable sensitivity only occurred in a small proportion of patients. Some patients who were initially responsive to the therapy eventually developed acquired resistance. Therefore, studying the mechanisms of anti-PD1/PDL1 resistance is critical for figuring out overcoming strategies.

In this review we summarized the mechanisms of primary and acquired resistance of anti-PD1/PDL1 therapy, which contributed to low response rates and limited durability in solid tumors. However, because of the complexity of antitumor immunity, the documented mechanisms are just pieces of the puzzle and varied within each individual, making it intricate for properly selecting patients and designing overcoming strategies. Currently, the efficiency of extensively used biomarkers for identifying responders is not stable, and most of the mechanism-based strategies are still undergoing preclinical studies while some of the clinical trials had been shut down because of severe adverse effects and limited benefits. Thus, picturing a more detailed map of resistant mechanisms, designing proper trials and exploring appropriate biomarkers are necessary for guiding precision therapy and expanding the spectrum of responders in the future.

## Author Contributions

QL and YZ conceptualized this review and decided on the content. QL wrote the manuscript. QL and KS prepared the figures and tables. YZ, DW, and LW revised this review. All authors approved the final version of the manuscript and agreed to be accountable for all aspects of the work.

## Conflict of Interest

The authors declare that the research was conducted in the absence of any commercial or financial relationships that could be construed as a potential conflict of interest.

## Abbreviations

APC: antigen presenting cell
CSF1R: colony-stimulating factor 1 receptor
CTLA4: cytotoxic T lymphocyte-associated antigen 4
DC: dendritic cell
ICB: immune checkpoint blockade
IDO: indoleamine 2,3-dioxygenase
IHC: immunohistochemistry
KYN: kynurenine
LAG3: lymphocyte activation gene 3
LCMV: chronic lymphocytic choriomeningitis virus
LOH: loss of heterozygosity
MAPK: mitogen-activated protein kinase
MDSC: myeloid-derived suppressor cell
MHC: major histocompatibility complexes
NSCLC: non–small cell lung cancer
PD1: programmed cell death 1
PDL1: programmed cell death ligand 1
PI3K: phosphatidylinositol-3-kinase
PTEN: phosphatase and tensin homolog
STAT: signal transducers and activators of transcription
TAM: tumor associated macrophage
TGF-β: transforming growth factor beta
TIL: tumor infiltrating lymphocyte
TIM3: T-cell immunoglobulin and mucin-domain containing 3
TMB: tumor mutation burden
TME: tumor microenvironment
TOX: thymocyte selection-associated high mobility group box gene
Treg: regulatory T cell
TRP: tryptophan.
